# Price and reimbursement of advanced therapeutic medicinal products in Europe: are assessment and appraisal diverging from expert recommendations?

**DOI:** 10.1186/s40545-021-00311-0

**Published:** 2021-03-19

**Authors:** Virginia Ronco, Myriam Dilecce, Elena Lanati, Pier Luigi Canonico, Claudio Jommi

**Affiliations:** 1 Market Access Provider Srl, Via V. Monti, 3, 20123 Milan, Italy; 2 Dipartimento di Scienze del Farmaco, Università del Piemonte Orientale, Largo Donegani 2, 28100 Novara, Italy; 3 SDA Bocconi School of Management, Università Bocconi, Via Sarfatti 10, 20136 Milano, Italy

**Keywords:** ATMPs, Pricing and reimbursement, HTA, Pipeline

## Abstract

**Background:**

Advanced therapy medicinal products (ATMPs) represent an important cornerstone for innovation in healthcare. However, uncertainty on the value, the high average cost per patient and their one-shot nature has raised a debate on their assessment and appraisal process for pricing and reimbursement (P&R) purposes. This debate led experts providing for recommendations on this topic. Our primary objective is to investigate the ATMPs P&R process in the main five European countries and to understand if this process is consistent with published P&R expert recommendations. We also investigated the current ATMP pipelines to understand if future ATMPs will create challenges for their P&R process.

**Methods:**

P&R framework for ATMPs in the European Major five (EU5) countries was investigated through a literature search on PubMed, institutional websites of National Health Authorities and grey literature. The ATMPs pipeline database was populated from a clinical trial database (clinicaltrials.gov), relying on inclusion and exclusion criteria retrieved from the literature.

**Results:**

Reimbursement status of ATMPs is different across the EU5 countries, with the exception of CAR-Ts which are reimbursed in all countries. Standard P&R process in place for other medicinal products is extended to ATMPs, with the exception of some cases in Germany. List prices, where available, are high and, tend to be aligned across countries. Outcome-based Managed Entry Agreements (MEAs) have been extensively used for ATMPs. Extra-funds for hospitals managing ATMPs were provided only in Germany and, as additional fund per episode, in France. The accreditation process of hospitals for ATMPs management was in most countries managed by the national authorities. As far as ATMPs pipeline is concerned, ATMPs in development are mostly targeting non-rare diseases.

**Conclusions:**

Expert recommendations for ATMPs P&R were partially applied: the role of outcome-based MEAs has increased and the selection process of the centres authorized to use these treatments has been enhanced; additional funding for ATMPs management to accredited centres has not been completely considered and annuity payment and broader perspective in cost considerations are far from being put in place. These recommendations should be considered for future P&R negotiations to pursue rational resource allocation and deal with budget constraints.

## Background

Advanced therapy medicinal products (ATMPs), defined by Directive 2001/83/EC, integrated by Regulation 1394/2007, include gene therapies, somatic-cell therapies, tissue-engineered medicines, and products containing one or more medical devices (combined ATMPs) [1].

ATMPs assessment and appraisal issues [2], in particular gene therapies [3–6], and implications on the Pricing and Reimbursement (P&R) process have been recently investigated.

ATMPs are often used to treat severe diseases associated with considerable societal costs [3, 4].

Clinical trials are characterized by small patient populations, short investigational duration and single-arm trial design. This is supportive in accelerated procedures granted to medicines for rare diseases with a high unmet need but creates problems for Health Technology Assessment (HTA) [3, 5]. ATMPs are one-shot costly therapies, whose benefits can only be appreciated in a longer-term perspective. The temporal misalignment between incremental and avoided costs represents a challenging issue for budget constraints; payers may focus on the short-term economic impact of medicines, disregarding saving in the long-run [6]. Despite the overall impact on budget being similar to that of a cheaper treatment for a larger patient population, the emotional impact of high prices is stronger and may undermine the principle of equity [4]. In addition, ATMP management and administration is complex and requires: (i) a clear definition of centres of excellence with high-quality equipment and expertise of health-care professionals; (ii) proper funding of the centres, and (iii) facilitated patient access to effective new therapies [6].

These issues have been widely discussed by the literature, driving various recommendations by experts on the assessment and appraisal of ATMPs. In general, experts have not supported a specific value framework for ATMP, but they have recommended to (i) collect more robust evidence [6]; (ii) increase awareness on the value of ATMPs in order to overcome prejudice or excessive unjustified optimism [6]; (iii) strengthen the early dialogue between HTA authorities, payers and other stakeholders, including patients [6]; (iv) conduct post-marketing assessment programmes (which may include registries or observational studies) with the purpose to gain additional information on the efficacy and safety profile of ATMPs [6]; (v) rely on outcome-based Managed Entry Agreements (MEAs) to manage uncertainty on benefits and on annuity payment to split treatment costs over time [6]; (vi) work on an early definition of the criteria for a proper identification of the centres of excellence [6]; and (vii) to adopt a societal perspective in the economic evaluation of ATMPs (or at least a double reference case approach) and face challenges posed by ATMPs to cost–effectiveness analysis, including discount rates and modelling [3, 5].

The primary objective of this paper is to investigate how the P&R of ATMPs has been managed in the European Major five (EU5) countries and whether it is consistent with published expert recommendations. We also compared the current ATMP pipelines with already marketed treatments with the aim of identifying the issues that may be challenging in the P&R process in the future.

## Materials and methods

A literature review on the ATMPs P&R status and regulatory frameworks across EU5 was conducted using PubMed (as scientific literature source), the institutional websites of the National Health Authorities for each of the five EU countries [7–14], and grey literature (google and non-peer review journals) using as cut-off date July 2020. ATMPs reimbursement status was defined as “reimbursed ATMP”, “not reimbursed ATMP” or “ongoing evaluation” and was assessed searching the institutional websites.

For pipeline analysis interventional clinical trials on ATMPs were retrieved from the clinicaltrials.gov database (www.clinicaltrials.gov) with a temporal limitation from July 2017 to November 2019. Hanna et al. 2016 keywords were used for a preliminary screening of ATMPs trials (i.e. trials focused on ATMPs) [15]. This first screening was validated using Committee for Advanced Therapies (CAT) criteria [16]. Only trials that met inclusion and exclusion criteria described in Table 1 were considered.

**Table 1:** Database inclusion and exclusion criteria

*Inclusion criteria*	
Study type	Interventional
Phase	II, II/III and III
Keywords	From Hanna et al. 2016 [15]
Study status	Ongoing or completed
Last update	1/07/2017–1/7/2019
ATMPs definition	As per Directive 2001/83/EC, modified by Regulation 1394/2007 and Committee for Advanced Therapies algorithm [16]
*Exclusion criteria*	
Study type	Non-interventional, observational, patient registries, expanded access
Phase	Early I, I, IV, and not applicable
Study status	Unknown, withdrawn, suspended, terminated

Rarity of the targeted diseases in each trial was searched on the Orphanet website [17]. Based on the European Medicine Agency (EMA) definition [18], diseases were considered rare if their prevalence does not exceed 5 cases in 10,000 people. An Excel® 2010 extraction template (Microsoft Corporation) was created to include all data for each trial identified: ATMP, Completion Date, Diseases, Rarity of the disease, First Posted, Funded By, Interventions, Last Update Posted, Locations, NCT (Number of Clinical Trial), Outcome, Phases, Primary Completion Date, Results, Sponsor/Collaborators, Start Date, Status, Study Results, Study Type, Therapeutic area, Title, URL. Duplicates and trials on investigational products other than ATMPs were excluded and finally the database was analysed according to ATMPs type, trial status, funding origin, therapeutic area and disease rarity (Table 2).

**Table 2:** Domains considered in the database analysis

Analysis domains	
ATMP type	Gene therapy
	Cell therapy
	Tissue engineered product
Clinical trial status	Recruiting
	Ongoing
	Completed
	Active, not yet recruiting
Funding origin	Company
	Other
	Co-funding
Therapeutic area	Oncological disorders
	Onco-haematological disorders
	Cardiovascular disorders
	Immunological disorders
	Cartilaginous–musculoskeletal disorders
	Gastrointestinal disorders
	Metabolic disorders
	Haematological disorders
	Ophthalmological disorders
	Neuro-neuromuscular disorders
	Other disorders
Disease rarity	Rare
	Not rare

## Results

The following two sections describe the current P&R process for ATMPs in EU5 countries and the pipelines. Consistency of the former with the recommendations of the literature and problems posed by the latter to future P&R negotiations will be discussed afterwards.

### P&R process for ATMPs in EU5 countries

At the time of the analysis (July 2020), 15 ATMPs achieved marketing authorization (MA) in the European Union (EU); of these, ten have received orphan designation, ten have an active MA (Holoclar, Imlygic, Strimvelis, Spherox, Alofisel, Kymriah, Yescarta, Luxturna, Zynteglo, and Zolgensma) and five had already been withdrawn from the market for commercial reasons (ChondroCelect, Glybera, Provenge, Maci, and Zalmoxis) [19–33]. Despite being recently withdrawn from the market a few years after obtaining MA, Zalmoxis was included in the analysis because of its P&R assessment by all EU5 countries. After MA is granted, the Marketing Authorisation Holder (MAH) has to submit a P&R request in each country to ensure patient access of a new ATMP.

The P&R request was not submitted for all ATMPs in each of the EU5 countries: (i) no request for Imlygic submission in France, Spain and Italy; (ii) no submission for Strimvelis in France; (iii) no submission for Spherox in Italy and France (Table 3).

**Table 3:** ATMPs status across EU5

Country	Italy			UK			France			Germany			Spain		
ATMP	Status	MEAs	Price, discounts	Status	MEAs	Price, discounts	Status	ASMR/SMR	Price, discounts	Status	NUB, MEAs	Price	Status	MEAs	Price
Holoclar	✓	Payment by result	95,000 €	✓ STA, restricted target population	PAS	88,993 €/eye (hidden discount excluded)	✓	ASMR IV	Reimbursed as DRG	✓ Medical procedure	NUB 4	DRG price	✗	–	–
Imlygic	✗ Request not submitted	–	–	✓ STA	PAS	1.858 €/vial (hidden discount excluded)	NA	–	–	✓ No additional benefit	NUB 1	Lauertaxe at launching: 2,398.50 €. After negotiation: 1,220.52€	✗ Request not submitted	–	–
Strimvelis	✓ Innovative status (expired)	Payment by result	594,000 €	✓ HST		594,000 € (+ hospital costs coverage in Italy)	NA	–	–	✗	–	–	✗	–	–
Zalmoxis*	✓	Flat price/patient	149,000 €	NA	–	–	✗	SMR insufficient	–	✓ Not quantifiable benefit	NUB 1	Lauertaxe at launching: 163,900€. After negotiation: 130,000€	✗	–	–
Spherox	✗ Request not submitted	–	–	✓ STA, Restricted target population	–	11,124 € (hidden procurement discount excluded)	NA	–	Reimbursed as DRG	✓ Medical procedure	–	–	✗	–	–
Alofisel	✗	–	–	✗	–	Submitted price: 60,083 €	✓	ASMR IV	54,000 €	✓ Not quantifiable benefit	NUB 1	60,000 €	✓	–	60,000 €/ treatment
Kymriah	✓ Innovative status for both indications	Payment "at" result for both indications	320,000 €, discount for DLBC	✓ STA	CDF (MAA, CAA)	313,766 € (hidden discount excluded)	✓	ASMR IV for DLBCL; ASMR III for ALL	297,666 € + 15,000 € on top of DRG	✓ Not quantifiable benefit (re-assessment after 1 year)	NUB 1; Outcome-based MEA with few insurance	320,000 €	✓	Payment "at" result for both indications	320,000 €
Yescarta	✓ Innovative status for both indications	Payment "at" result for both indications	327,000 €, discount	✓ STA	CDF (MAA, CAA)	Confidential price + discount	✓	ASMR III	327,000 € + 15,000 € on top of DRG	✓ Not quantifiable benefit (re-assessment after 3 year)	NUB 1; Outcome-based MEA with few insurance	327,000 €	✓	Payment "at" result for both indications	327,000 €
Luxturna	Ongoing CPR	–	–	✓ HST	–	682,673 € (hidden discount excluded)	Ongoing	ASMR II	–	✓ Considerable added benefit	NUB 1	345,000 €	Ongoing	–	–
Zynteglo	Ongoing CTS	–	–	Ongoing STA	–	–	Ongoing	ASMR III	–	✓ Not quantifiable added benefit	315,000 € up front and 4 additional annual payments (at result)	1.58 million € (full price)	Ongoing	–	–
Zolgensma	Ongoing CTS	–	–	Ongoing HSTP	–	–	✓ ATU	–	NA	Ongoing	–	–	Ongoing	–	–

*ALL* acute lymphoblastic leukaemia; *ASMR* Amélioration du Service Médical Rendu (additional clinical value); *ATU* Autorisations Temporaires d'Utilisation (temporary authorization for use); *CAA* Commercial Access Agreement; *CPR* Price and Reimbursement Committee; *CTS* Scientific Technical Committee; *DLBCL* diffuse large B-Cell lymphoma; *HST* Highly Specialized Technologies; *MAA* Market Access Agreement; *MEA* Managed Entry Agreement; *NA* not available; *NUB* Neue Untersuchungs und Behandlungsmethode (new examination and treatment method); *PAS* Patient Access Scheme (mainly discounts); *P&R* Price and Reimbursement; *SMR* Service Médical Rendu (absolute clinical value); *UK* United Kingdom

*Withdrawn. ✓ = reimbursed; ✗ = not reimbursed

All ATMPs were assessed as medicines and followed the “traditional” appraisal procedure in each country, except for Germany. In Germany, the Federal Joint Committee (G-BA) first categorizes the ATMP either as a medicine or a medical procedure. If the ATMP has pharmacologic properties and its clinical outcome is not dependent on the healthcare professional skills, it is categorized as a medicine and undergoes the benefit assessment procedure according to the AMNOG (*Arzneimittelmarktneuordnungsgesetz*, Medicine Market Reorganization Act), where discounts on list prices determined by the MAH are negotiated on the grounds of different variables, including the added therapeutic value. Otherwise, it is categorized as a medical procedure and is normally assessed by the PEI (Paul-Ehrlich-Institut) [34–37]. Spherox and Holoclar were evaluated via the medical procedure; all other ATMPs underwent the AMNOG process (Table 3).

In United Kingdom, the National Institute for Health and Care Excellence (NICE) evaluates ATMPs through two different HTA processes: the STA (Single Technology Appraisal) or the HSTP (Highly Specialised Technologies Programme). The STA is used for products targeting non-rare diseases and relies on cost–effectiveness analysis (CEA); the HSTP evaluates products for ultra-rare diseases, allowing a higher Incremental Cost–Effectiveness Ratio (ICER) threshold [38]. Among the most recently appraised ATMPs, Kymriah and Yescarta have been evaluated through STA, while Strimvelis and Luxturna by HSTP. Zynteglo and Zolgensma are currently under assessment through STA and HSTP, respectively. Alofisel received a negative recommendation.

In France, medicinal products are assessed and appraised by the Transparency Committee (TC) of the Haute Autorité de Sante (HAS). If the Service Médical Rendu (SMR, absolute clinical value) of a drug is considered sufficient, the medicine can be granted reimbursement and then evaluated for its *Amélioration du Service Médical Rendu* (ASMR, additional clinical value). ASMR is a driver for price negotiation with the *Comité Économique des Produits de Santé* (CEPS, Committee for Healthcare Products). The ASMR rating, assigned by TC, ranges from I (maximum, revolutionary therapy) to V (minimum, no superiority *versus* standard of care) [39–41]. Patient access to a new product is possible also before MA with the Authorization for Temporary Use (ATU). To date, Kymriah, Yescarta, and Zolgensma obtained ATU; the ATU for Zolgensma is the only one currently active. During the ATU period, the MAH is allowed to set a launch price before the negotiation with the CEPS.

In Spain, the *Agencia Española de Medicamentos y Productos Sanitarios* (AEMPS) performs a clinical assessment of new medicinal products and then the General Directorate for Medicines of MoH (*Dirección General de Cartera Básica de Servicios del Sistema Nacional de Salud y Farmacia*, DGCBSF) prepares a price proposal. Negotiation of the proposal occurs between the Price Committee (*Comisión Interministerial de Precios de Medicamentos y Productos Sanitarios*, CIPM) and the MAH [42]. The final price is the maximum reimbursable price nationwide, subject to further reduction by hidden discounts negotiated by the Regions [43], due to the fact that the Spanish Healthcare System is decentralized and each Region has the authority to re-negotiate the prices.

In Italy, the assessment and appraisal process is managed by the Italian Medicines Agency (*Agenzia Italiana del Farmaco*, AIFA), through its Scientific Technical Committee (*Commissione Tecnico Scientifica*, CTS) and its Price and Reimbursement Committee (*Comitato Prezzi e Rimborso*, CPR). The CTS evaluates the clinical added value, the place in therapy of the new medicine and possible price comparators. In the case of a positive opinion, the CPR negotiates with the MAH the price and any MEAs [44]. If the product is reimbursed and the price is negotiated at a national level, a request for inclusion in the regional and/or hospital therapeutic lists has to be submitted. The MAH may also ask for the recognition of the “innovative status” of the medicinal product. Innovativeness is appraised on the grounds of three criteria: unmet medical need, added therapeutic value and quality of available evidence and lasts maximum 3 years [45]. Innovative medicines benefit from dedicated funds and immediate access to regional lists, and they are not subject to temporary price reductions by law. Currently, only three ATMPs (Strimvelis, Kymriah, and Yescarta), have been granted this status (for Strimvelis the innovativeness status has expired).

Only Chimeric Antigen Receptor-T cells (CAR-Ts) therapies are reimbursed in all the EU5 while the decisions regarding other products are quite heterogeneous (Table 3).

List prices, on the other hand, are substantially aligned across all the EU5 (Table 3), despite in Italy, Spain and France they are negotiated together with the reimbursements status, whereas in Germany and England prices are freely determined by the relevant MAH, but indirectly regulated (in Germany a discount is negotiated after 1 year of marketing; in England a threshold range for the incremental cost–effectiveness is set, thus addressing pricing strategies by the pharmaceutical companies).

ATMPs were also quite disruptive for MEAs trends in almost all the EU5. Outcome-based agreements, in the form of performance-linked reimbursement, had never been signed centrally in Spain before a “payment at result” for the two CAR-Ts; in Italy, a similar agreement as the one above for the two CAR-Ts was signed, despite the outcome-based agreements being gradually substituted by simple discounts. An instalment plan, with 315,000 € paid up front and four additional annual payments only if the treatment continues to be effective, was applied for Zynteglo in Germany [46]. In England, NICE has created a Cancer Drug Fund (CDF), as a time-limited managed access agreement, which guarantees a faster and temporary reimbursement for cancer medicines, conditioned by evidence-based results which allow a routine appraisal. The two CAR-Ts were included in the CDF, due to the uncertainty of their benefit and cost–effectiveness profiles.

Germany and France adopted extra-cost coverage systems to support the management of ATMPs in hospitals. In Germany, when a medicine is used in a hospital setting and its price is not totally covered by an existing diagnosis-related group (DRG) fee, it is possible apply for an extra-budgetary reimbursement, the NUB (*Neue Untersuchungs- und Behandlungsmethoden*, new examination and treatment method). A status of NUB 1 is granted to a medicine or procedure which is new, innovative, for a low number of patients and which requires higher resource use (other than that covered by the DRG); this means that an extra-coverage can be negotiated by hospitals [47, 48]. In France, an additional 15,000€ (fixed) was added to the current DRG fee exclusively for Kymriah and Yescarta (Table 3).

As far as other polices that affect patients access to ATMPs, in most cases, the accreditation criteria for centre selection are established by the National Authorities, usually through the JACIE (Joint Accreditation Committee ISCT-Europe and EBMT) accreditation process and with the involvement of multidisciplinary medical teams [49–53]. In England, the National Health Service has been working with the JACIE and life sciences companies to get centres up and running. In Spain, for CAR-T, a group of experts for the definition of criteria for designation of centres was established. In Italy, the accreditation process is usually managed by the regional governments, regulated by the minimum criteria for centres' selection drawn up by AIFA for ATMPs.

### Pipeline analysis

According to the previously mentioned inclusion and exclusion criteria, 6,982 trials were identified. After the exclusion of duplicates and all studies not targeting ATMPs, 249 clinical trials were included in the final analysis.

**Fig. 1:**
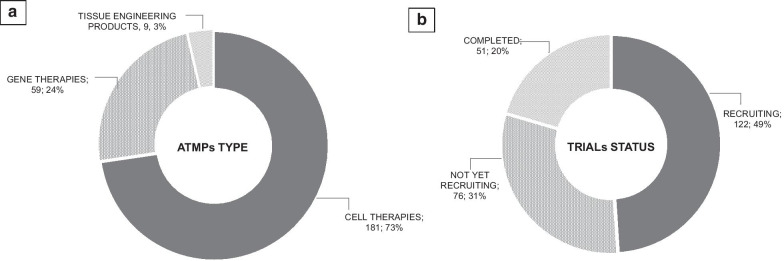
Clinical trial analysis according to type (**a**) and status (**b**). The current ATMP pipeline analysed retrieved clinical trials on ATMPs from the clinicaltrials.gov database and selecting with the following inclusion criteria: interventional studies, phase II/, II/III and III studies, ongoing or completed studies, 1/07/2017–1/7/2019 as temporal limitation, and Directive 2001/83/EC plus Committee for Advanced Therapies algorithm used as definition of “ATMP”. Of 249 clinical trials included in the final analysis (after exclusion of duplicates and those not targeting ATMPs), 181 (73%) were conducted on cell therapies (CAR-Ts included), 59 (24%) on gene therapies, and 9 (4%) on tissue engineering products (figure **a**), while 122 (49%) were actively ongoing and recruiting, 76 (31%) not yet recruiting, and 51 (20%) completed (figure **b**)

Out of these, 181 (73%) are conducted on cell therapies (CAR-Ts included), 59 (24%) on gene therapies, and 9 (4%) on tissue engineering products. One hundred and twenty-two (49%) trials are actively ongoing and recruiting, 76 (31%) not yet recruiting, and 51 (20%) have been completed (Fig. 1).

**Fig. 2:**
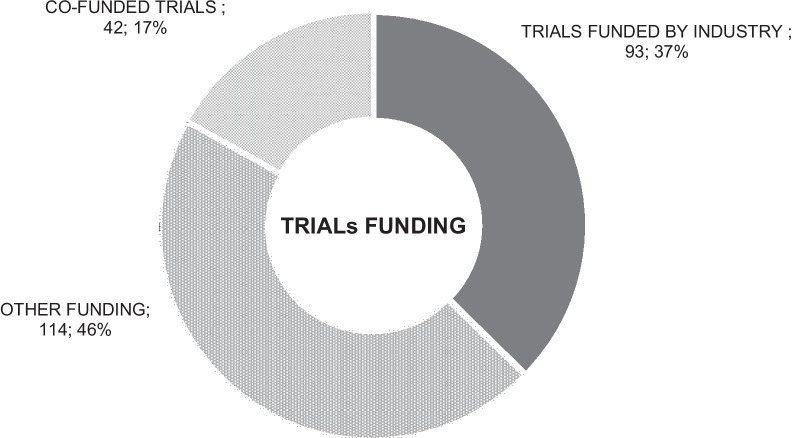
Funding of clinical trials with ATMPs. The current ATMP pipeline analysed retrieved clinical trials on ATMPs from the clinicaltrials.gov database and selecting with the following inclusion criteria: interventional studies, phase II/, II/III and III studies, ongoing or completed studies, 1/07/2017–1/7/2019 as temporal limitation, and Directive 2001/83/EC plus Committee for Advanced Therapies algorithm used as definition of “ATMP”. Of 249 clinical trials included in the final analysis (after exclusion of duplicates and those not targeting ATMPs), 93 (37%) were funded by companies, 114 (46%) through other sources, and 42 (17%) were co-funded by companies and others

Ninety-three trials (37%) were funded by companies, 114 (46%) through other sources, and 42 (17%) were co-funded by companies and other organizations (Fig. 2).

The main target of ATMPs under development are oncological and onco-haematological diseases (110; 44%), followed by cardiovascular (35; 14%) and immunological (17; 7%) diseases.

**Fig. 3:**
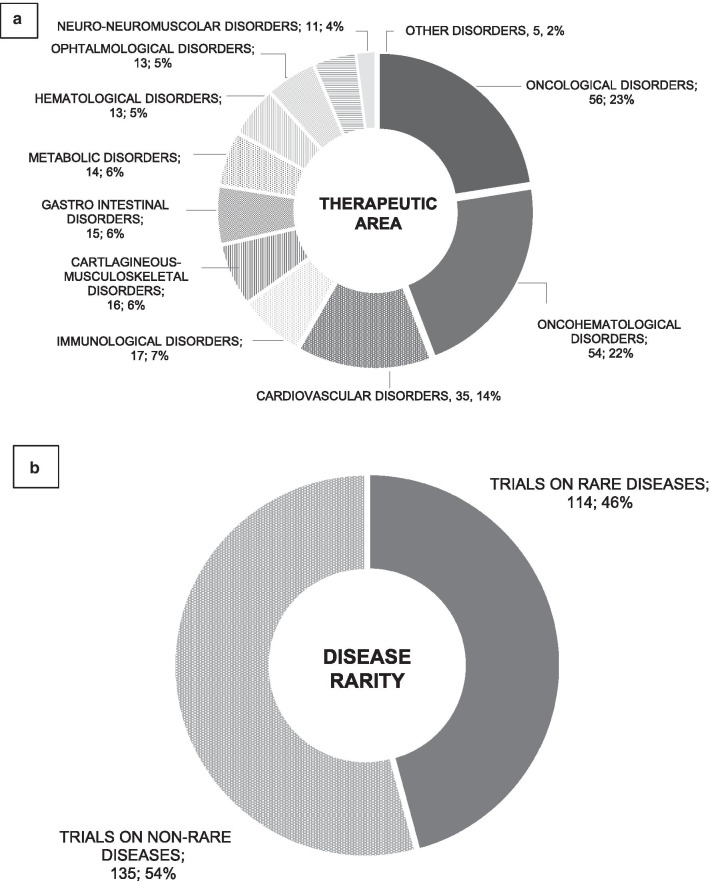
Therapeutic area (**a**) and disease rarity (**b**) of ATMPs clinical trials. The current ATMP pipeline analysed retrieved clinical trials on ATMPs from the clinicaltrials.gov database and selecting with the following inclusion criteria: interventional studies, phase II/, II/III and III studies, ongoing or completed studies, 1/07/2017–1/7/2019 as temporal limitation, and Directive 2001/83/EC plus Committee for Advanced Therapies algorithm used as definition of “ATMP”. Of 249 clinical trials included in the final analysis (after exclusion of duplicates and those not targeting ATMPs), 110 (44%) were on oncological and onco-haematological diseases, 35 (14%) on cardiovascular diseases and 17 (7%) on immunological diseases (figure **a**), while 114 (46%) on a rare disease, and the remaining 135 (54%) on not rare one (figure **b**)

The study target was a rare disease in only 114 (46%) trials, while the target disease was not rare in the remaining 135 (54%) (Fig. 3).

## Discussion

This paper aimed at comparing assessment, appraisal and P&R process for ATMPs in the main European countries and whether this process is consistent with the expert recommendations currently available.

This study shows similar common patterns. ATMPs follow the same procedure already in place for other medicinal products in all countries, with the exception of Germany. Since the medicines P&R process is different across countries, this makes the reimbursement status of ATMPs heterogenous, with the exception of CAR-T therapies, which have been granted reimbursement in all countries due to the high expectations of their clinical benefits. Furthermore, for some ATMPs, request for P&R was not submitted in all countries. List prices tend to remain aligned in order to avoid cross-reference pricing. Our results also indicate that outcome-based MEAs and discounts are extensively used due to the uncertainty on the risk–benefit profile of ATMPs and their sustainability over time, respectively. This makes net prices divergent from list prices. Comparison of discounts on net prices is not possible as they are not published in any of the EU5 countries, with the exception of Germany. Additional funding for managing ATMPs was provided only in Germany (through the ordinary process of NUB classification) and in France (15,000 € for each DRG) for CAR-Ts alone.

Assessment and appraisal for P&R are partially aligned with the expert recommendations. Payers show a higher availability to draw up outcome-based MEAs, centrally or locally. On the other hand, other expert recommendations have not been applied systematically: ad hoc additional coverage for ATMP administration and patient management was provided only in France, with limited funding. The two delayed payments at results MEAs in Spain and Italy differ from each other both in terms of payment schemes as well as evaluated overall outcome. To date, no country has applied annuity payments, apart from Germany (and one product). We did not find concrete evidence of anticipated early dialogue among stakeholders or a more holistic evaluation of the social impact of these diseases considering the avoided productivity loss.

Pipeline analysis showed that the role of ATMP-based therapy is rapidly evolving from a niche-based setting in rare diseases to a vaster application in diseases which involve larger target populations. In this potential scenario of aiding a larger population, the impact on budget will be higher, if unitary prices remain very high as they are for current ATMPs.

## Conclusions

The P&R process for ATMPs in the largest European countries is similar to the one adopted for other medicines. This is aligned to what experts have recommended, i.e. not creating an “ad hoc” framework for P&R of ATMPs, but adapting the assessment and appraisal processes for medicines to ATMPs.

However, some recommendations have not been sufficiently pursued, by domestic payers, including the suggestion of: (i) promoting early dialogue among HTA bodies, payers, the industry, and other stakeholders; (ii) considering, more than for other medicines, the long-term impact and using societal perspective in determining value for money and impact on budget; (iii) introducing annuity payment schemes, which would mean to actually turn the current expenditure into investment.

For the future, since many new ATMPs are going to be launched for non-rare diseases, it is important (i) evaluating in advance the organizational impact of ATMPs and providing health-care centres with the necessary resources; (ii) estimating the budget impact of ATMPs through an appropriate horizon scanning activity; and (iii) implementing a price/volume trade-off strategy or prioritizing patients who can benefit more from treatment according to clinical data.

## Data Availability

Not applicable.

## Abbreviations

AEMPS: Agencia Española de Medicamentos y Productos Sanitarios
AIFA: Agenzia Italiana del Farmaco
AMNOG: Arzneimittelmarktneuordnungsgesetz
ASMR: Amélioration du Service Médical Rendu
ATMPs: Advanced Therapy Medicinal Products
ATU: Authorization for Temporary Use
CAR-T: Chimeric Antigen Receptor-T cell
CAT: Committee for Advanced Therapies
CDF: Cancer Drugs Fund
CEA: Cost-Effectiveness Analysis
CEPS: Comité économique des produits de santé
CIPM: Comisión Interministerial de Precios de Medicamentos y Productos Sanitario
CPR: Comitato Prezzi e Rimborso
CTS: Commissione Tecnico Scientifica
DGCBSF: Dirección General de Cartera Básica de Servicios del Sistema Nacional de Salud y Farmacia
DRG: Diagnosis-Related Group
EMA: European Medicine Agency
EU: European Union
EU5: European Major five (Germany, United Kingdom, France, Spain, Italy)
HAS: Haute Autorité de Sante
HSTP: Highly Specialised Technologies Programme
HTA: Health Technology Assessment
ICER: Incremental Cost–Effectiveness Ratio
IPT: Informe de Posicionamiento Terapéutico
JACIE: Joint Accreditation Committee ISCT-Europe and EBMT
MA: Marketing Authorization
MAH: Marketing Authorisation Holder
MEA: Managed Entry Agreement
MoH: Ministry of Health
NICE: National Institute for Health and Care Excellence
NUB: Neue Untersuchungs—und Behandlungsmethoden
P&R: Pricing and Reimbursement
PEI: Paul-Ehrlich-Institut
SMR: Service Médical Rendu
STA: Single Technology Appraisal
TC: Transparency Committee
